# Nonsteroidal Anti-Inflammatory Drugs (NSAIDs): Progress in Small Molecule Drug Development

**DOI:** 10.3390/ph3051530

**Published:** 2010-05-14

**Authors:** Praveen P. N. Rao, Saad N. Kabir, Tarek Mohamed

**Affiliations:** School of Pharmacy, Health Sciences Campus, University of Waterloo, 200 University Avenue W. Waterloo, ON, N2L 3G1 Canada

**Keywords:** Small molecules, NSAIDs, Lp-PLA_2_, mPGES-1, TNF-α

## Abstract

Ever since the discovery of aspirin, small molecule therapeutics have been widely prescribed to treat inflammation and pain. Aspirin and several small molecule NSAIDs are known to inhibit the enzymes cyclooxygenase-1 (COX-1) and -2 (COX-2). Despite the success of NSAIDs to treat inflammatory disorders, the development of a clinically useful small molecule NSAIDs with decreased side effect profiles is an ongoing effort. The recent discovery and development of selective COX-2 inhibitors was a step toward this direction. Emerging trends are represented by the progress in the development of hybrid agents such as nitric oxide donor-NSAIDs (NO-NSAIDs) and dual COX/lipoxygenase (LOX) inhibitors. This review focuses on the recent advances in the rational design of small molecule NSAIDs in therapy.

## 1. Introduction

The history of treating fever, pain and inflammation is a fascinating tale of human adventure that goes back centuries [1]. Since the discovery and isolation of salicin from willow bark in the early 18th century to the development of selective COX-2 inhibitors in the 1990s, small molecule therapies to treat fever, pain and inflammation have evolved [1,2]. Traditional NSAIDs such as aspirin (**1**), ibuprofen (**2**) and diclofenac (**3**) that exhibit nonselective COX inhibition represent some of the most widely prescribed NSAIDs to relieve short term fever, pain and inflammation [3,4]. The characteristic feature of these traditional nonselective COX inhibitor NSAIDs was the presence of a carboxylic acid (COOH) functional group. In the early 1990s the second isoform of COX was discovered, providing a novel target to develop anti-inflammatory agents with superior safety profiles compared to traditional NSAIDs [5,6]. Consequently, selective COX-2 inhibitors (coxibs) based on a diarylheterocyclic ring template as in celecoxib (**4**) and rofecoxib (**5**) were developed [7,8].

**Figure 1:**
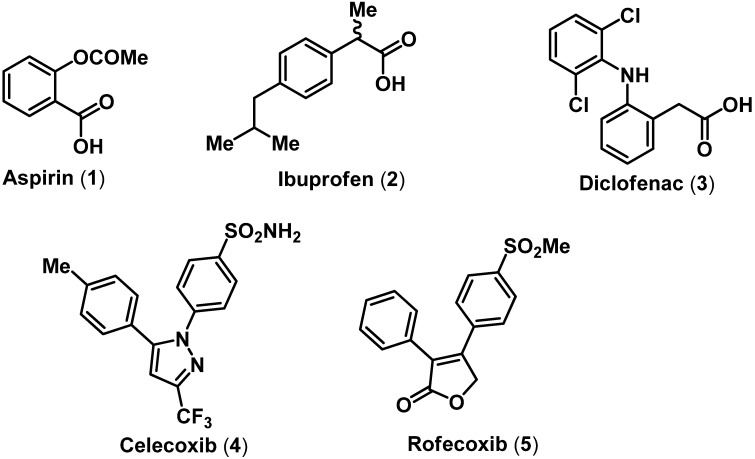
Chemical structures of some nonselective and selective COX inhibitors.

**Figure 2:**
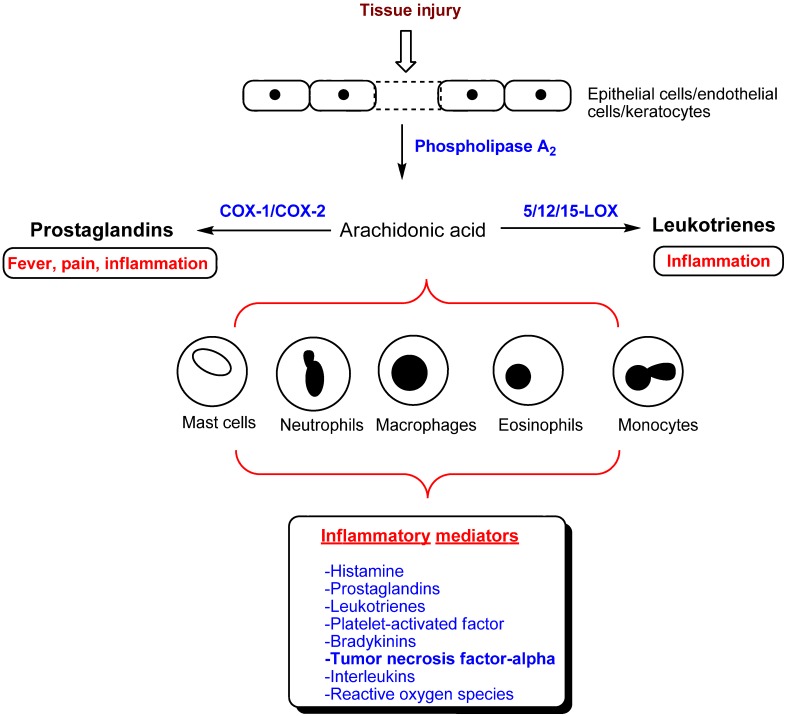
A simplified diagram of the inflammatory pathway and its mediators.

These agents were characterized by the presence of a *para*-sulfonamide (SO_2_NH_2_) or a *para*-methanesulfonyl (SO_2_Me) pharmacophore present on one of the aryl rings. Crystal structure studies supported the hypothesis that the *p*-SO_2_NH_2 _ or *p*-SO_2_Me pharmacophore was conferring COX-2 selectivity by orienting in a secondary pocket accessible only in the COX-2 active site [9,10]. The initial euphoria surrounding the selective COX-2 inhibitors, was short lived as studies indicated serious risks of cardiovascular complications in susceptible population during therapy [11,12]. Therefore, developing novel orally active small molecule anti-inflammatory agents with superior safety profile presents a significant challenge. The inflammatory pathway is a complex event involving multiple effectors (Figure 2). Inflammatory mediators such as prostaglandins (PGs), leukotrienes (LTs) and tumor necrosis factor-alpha (TNF-α) are implicated in a wide variety of diseases such as rheumatoid arthritis (RA), osteoarthritis (OA), asthma, atherosclerosis, different types of cancers and diseases of the central nervous system [13,14,15,16,17,18,19,20,21,22]. Traditional NSAIDs targeted COX isozymes, whereas later studies investigated nitric oxide (NO) donating NSAIDs (NO-NSAIDs), dual COX/LOX inhibitors, leukotriene receptor antagonists and selective COX-2 inhibitors in an effort to develop anti-inflammatory agents with superior safety profile [5,15,23,24,25]. Emerging small molecule targets includes phospholipases (PLA_2_), microsomal prostaglandin E_2_ synthase (mPGES-1) and inhibition of TNF-α. This review will focus on the recent drug discovery efforts toward developing novel small molecule ring templates as NO-NSAIDs, selective COX-2, dual COX/LOX, lipoprotein-PLA_2_ (Lp-PLA_2_), mPGES-1 and TNF- α inhibitors. 

## 2. NO-NSAIDs

The concept of developing hybrid NO-NSAIDs was primarily conceived to decrease the gastrointestinal (GI) toxicities observed with traditional NSAID use. In the GI tract NO is known to exert its protective role by increasing the mucous secretion, mucosal blood flow and inhibition of neutrophil aggregation [24]. In addition, the recent controversy surrounding the cardiovascular side effects of selective COX-2 inhibitors, further supports the need to develop clinically useful NO-NSAIDs since NO is also known to exhibit beneficial effects on the cardiovascular system by inhibiting platelet aggregation and adhesion [11,26]. Accordingly, several studies focused on developing NO-NSAIDs based on the aspirin, naproxen and diclofenac ring templates. These agents contain organic nitrates or nitrosothiols as the NO-donor moiety [24,27,28]. 

Diazeniumdiolates (NONOates) represent a unique structural moiety that can be incorporated to develop NO-donating agents [29]. Recently, Knaus and coworkers described the design and synthesis of hybrid aspirin, ibuprofen and indomethacin derivatives coupled to diazeniumdiolates as novel NO-donating prodrugs. 

**Figure 3:**
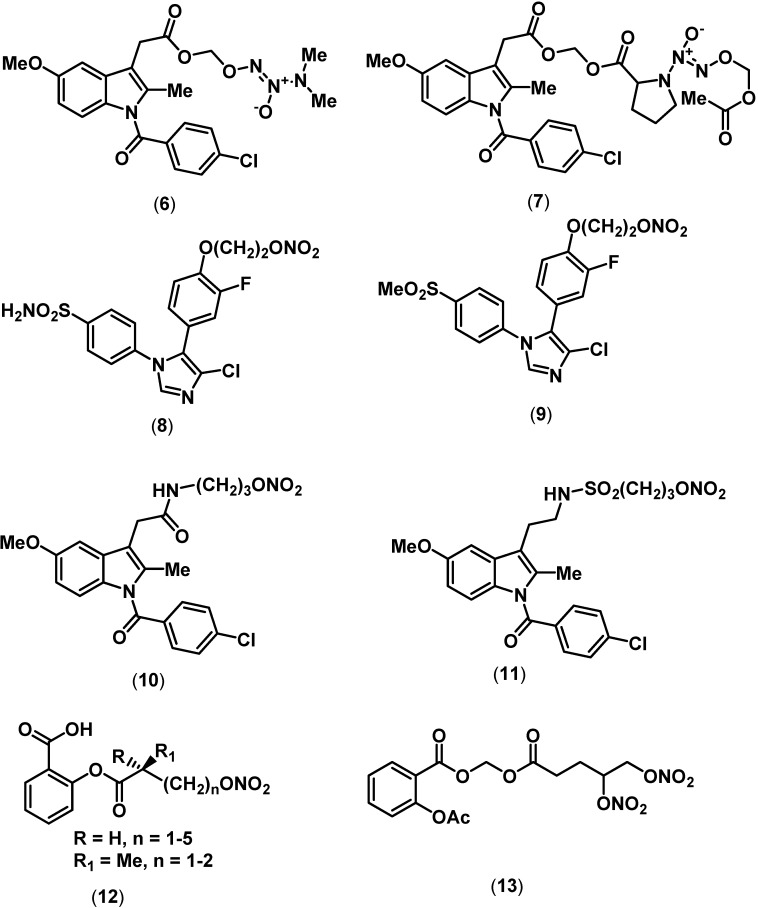
Chemical structures of some representative NO-donor anti-inflammatory agents.

The indomethacin derivative possessing a 1-(*N*,*N*-dimethylamino)diazen-1-ium-1,2-diolate (**6**, Figure 3) exhibited potent *in vivo* anti-inflammatory activity (ED_50_ = 5.9 mg/kg, oral dose) and minimal GI toxicity (ulcer index = 3.0 ± 0.3, oral dose) compared to the parent indomethacin (COX-2 IC_50_ = 5.7 µM; COX-1 IC_50_ = 0.10 µM). In another follow-up study, the indomethacin derivative linked to a 1-(2-carboxypyrrolidin-1-yl)diazen-1-ium-1,2-diolate by a methylene spacer (**7**) exhibited oral anti-inflammatory activity while exhibiting no GI toxicity (0.08 mmol/kg, oral dose). The *in vitro* assay data showed that these NO-donating prodrugs were devoid of COX inhibition [30,31]. 

Gasco and coworkers developed a series of metabolically stable selective COX-2 inhibitor NO-donors (CINODs) based on a 1,5-diarylimidazole ring template [32]. The organic nitrate, nitroxy-substituted alkyloxy groups were incorporated on one of the aryl rings as a NO-donor moiety. The sulfonamide derivative possessing a phenoxyethyl nitrate NO-donor moiety (**8**) retained COX-2 selectivity (COX-2 IC_50_ = 29 µM; COX-1 IC_50_ > 100 µM) and exhibited superior vasodilatory properties relative to the parent cimicoxib. The corresponding methanesulfonyl derivative (**9**) exhibited superior COX-2 selectivity (COX-2 IC_50_ = 6.9 µM; COX-1 IC_50_ > 100 µM) and vasodilatory properties relative to **8**. It should be noted that these novel CINODs exhibit decreased COX inhibitory potency relative to the lead selective COX-2 inhibitor cimicoxib (COX-2 IC_50_ = 0.10 µM; COX-1 IC_50_ = 1.9 µM). Recently, NitroMed Inc. reported a group of indomethacin derivatives as selective COX-2 inhibitors with NO-donating properties [33]. The indomethacin amide derivative possessing an organic nitrate NO-donor moiety (**10**, Figure 3) exhibited effective *in vitro* COX-2 selectivity (COX-2 IC_50_ = 1.2 µM; COX-1 IC_50_ = 6.0 µM) and oral anti-inflammatory activity. The sulfonamide derivative possessing a nitrooxypropyl NO-donor moiety (**11**) exhibited superior *in vitro* COX-2 selectivity relative to **10** and was an equipotent inhibitor of COX-2 with oral anti-inflammatory activity. However, only rats treated with **10** exhibited increased nitrite and nitrate concentration in plasma indicating its NO-donating property *in vivo*. In addition, **10** exhibited an 85% reduction in gastric lesions when administered orally in a rat model of aspirin-induced rat gastric damage model (dose = 45 µmol/kg).

The ever popular agent aspirin continues to be the focus of current research [34,35,36]. Recent studies have reported novel aspirin and aspirin derivatives possessing organic nitrate NO-donor moiety. In this regard, Gasco and coworkers prepared novel aspirin-like derivatives based on salicylic acid ring template possessing a nitrooxy-acyl NO-donor moiety (**12**) and aspirin derivatives possessing a (nitrooxyacyloxy)methyl ester NO-donor moiety (**13**). These agents exhibited effective oral anti-inflammatory and vasodilatory properties with reduced GI toxicities [36].

## 3. Selective COX-2 Inhibitors

**Figure 4:**
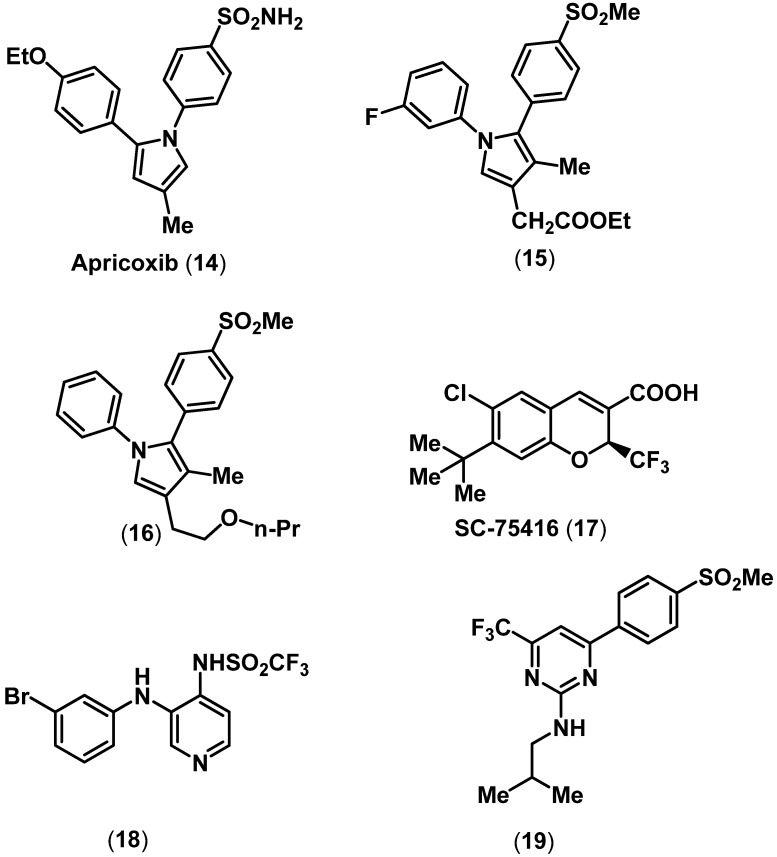
Chemical structures of some representative selective COX-2 inhibitors.

The adverse cardiovascular events associated with selective COX-2 inhibitors led to a dramatic decline in selective COX-2 inhibitor pipeline. In this regard, Tragara Pharmaceuticals is developing an orally active, selective COX-2 inhibitor (COX-2 IC_50_ = 0.31 µM; COX-1 IC_50_ = 2.2 µM) to treat different types of neoplasia such as tumors of lung, breast and pancreas [37]. Apricoxib has a 1,2-diphenyl template attached to a central 5-membered pyrrole ring along with a *para*-SO_2_NH_2_ COX-2 pharmacophore (**14**, Figure 4). In addition, a group of regioisomeric 1,5-diphenylpyrroles possessing a *para*-SO_2_Me COX-2 pharmacophore were reported as selective COX-2 inhibitors (**15**, COX-2 IC_50_ = 2.1 µM; COX-1 IC_50_ = 20.4 µM; **16** COX-2 IC_50_ = 0.018 µM; COX-1 IC_50_ > 100 µM) that exhibited effective oral anti-inflammatory and analgesic activities. However, these studies did not report the cardiovascular safety of these agents [38,39,40]. Furthermore, Pfizer scientists reported a group of benzopyran derivatives as selective COX-2 inhibitors [41]. These are structurally different from the diarylheterocyclic class of selective COX-2 inhibitors. A representative compound from this series SC-75416 (**17**) contains a COOH and a CF_3_ substituent. It is noteworthy that this agent did not contain either a SO_2_NH_2_ or a SO_2_Me COX-2 pharmacophore. Compound **17** exhibited effective oral anti-inflammatory activity and similar COX inhibition/selectivity (COX-2 IC_50_ = 0.25 µM; COX-1 IC_50_ = 49.6 µM) relative to celecoxib. 

In another study, Renard and coworkers designed novel nimesulide derivatives as selective COX-2 inhibitors [42]. The alkanesulfonamide (MeSO_2_NH) in nimesulide was replaced with a trifluoromethanesulfonamide (CF_3_SO_2_NH) moiety and the ether linkage was replaced with a secondary amine bridge. A representative agent **18 ** (Figure 4) exhibited a good combination of oral anti-inflammatory activity and *in vitro* COX-2 selectivity (COX-2 IC_50_ = 0.12 µM; COX-1 IC_50_ = 0.91 µM). In 2009, GlaxoSmithKline (GSK) scientists reported the development of a novel series of trifluoromethylpyrimidine based ring scaffolds (**19**, COX-2 IC_50_ = 206 nM; COX-1 IC_50_ = 62000 nM) as highly potent and selective COX-2 inhibitors [43,44]. Accordingly, several diverse classes of selective COX-2 inhibitors have been reported and a thorough discussion is beyond the scope of this review [45]. It should be noted that COX-1/COX-2 inhibition and selectivity data is highly variable based on the biochemical assay method used. In addition, *in vivo* anti-inflammatory/analgesic activities and side effects (GI, renal and cardiovascular) of NSAIDs are highly dose dependent. These factors contribute to the difficulty associated in determining specific COX-1 and COX-2 selectivity ratios for future development.

## 4. Dual COX/LOX Inhibitors

**Figure 5:**
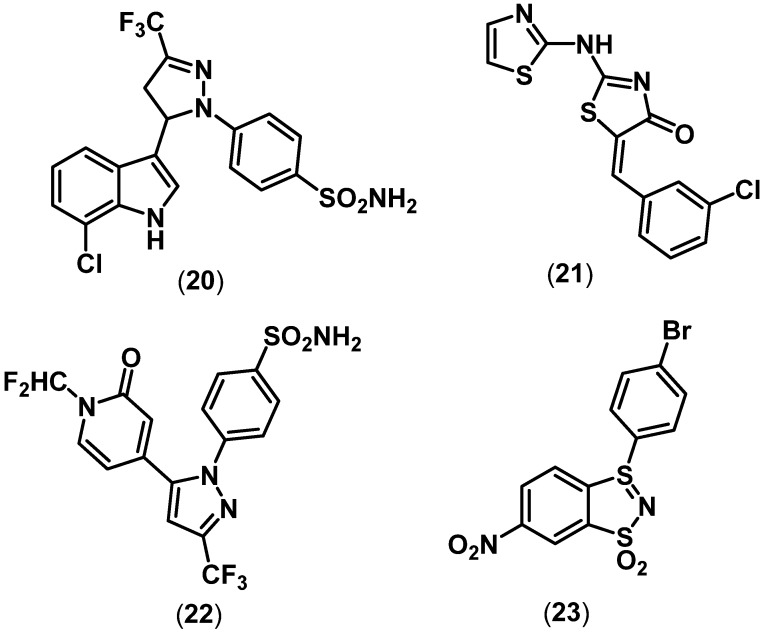
Chemical structures of some representative dual COX/LOX inhibitors.

Currently, LOXs are potential targets in the treatment of diseases such as asthma, atherosclerosis, cancer, and a variety of inflammatory conditions [14,15,16]. It was hypothesized that blocking the arachidonic acid (AA) metabolism via COX inhibition by either traditional NSAIDs or selective COX-2 inhibitors could lead to the generation of proinflammatory leukotrienes and lipoxins via the LOX pathway (Figure 2) partly accounting for the side effects seen with traditional NSAIDs and selective COX-2 inhibitors. To counter this, several dual small molecule COX/LOX inhibitors have been reported. Reddy and coworkers reported the development of racemic indolylpyrazoline class of agents as dual COX/LOX inhibitors. One of the examples shown in Figure 5 (**20**) had a *para*-SO_2_NH_2_ COX-2 pharmacophore and exhibited dual COX and LOX inhibition (COX-2 IC_50_ = 3.9 µM, COX-1 IC_50_ > 100 µM; 5-LOX IC_50_ = 94 µM, 12-LOX IC_50_ = 3 µM, 15-LOX IC_50_ = 36 µM). The levo isomers exhibit superior COX-2 inhibitory potency and selectivity. 

However, for this series of compounds, *in vivo* anti-inflammatory activities were not reported [46]. Furthermore, Lagunin and coworkers recently used structure-based virtual screening to identify suitable ring scaffolds as dual COX/LOX inhibitors [47]. This study revealed that a thiazolidinone ring scaffold could be used to develop novel anti-inflammatory agents. Compound **21** (Figure 5) exhibited weak *in vitro* COX and soyabean LOX inhibitory potency (COX-2 IC_50_ = 262 µM, COX-1 IC_50_ = 125 µM; LOX IC_50_ = 125.9 µM). *In vivo* **21** exhibited good anti-inflammatory activity (44.5% inhibition, dose = 0.01 mmol/kg) when administered through intraperitoneal route in animal models. However, oral activity was not reported. 

LOX isozymes are non-heme enzymes containing a catalytic ferric iron with a high reduction potential. Studies have indicated that iron chelators such as catechols and hydroxamic acid derivatives are capable of reducing the iron to its inactive state, thereby preventing the conversion of fatty acids to pro-inflammatory lipid mediators [48]. In this regard, Knaus and coworkers developed several hybrid COX/5-LOX inhibitors possessing a novel *N*-difluoromethyl-1,2-dihydropyridine-2-one LOX pharmacophore [49,50,51]. It was anticipated that the CONCHF_2_ moiety present in *N*-difluoromethyl-1,2-dihydropyridine-2-one acts as a cyclic hydroxamic acid mimetic by chelating LOX iron. A celecoxib derivative incorporating the *N*-difluoromethyl-1,2-dihydropyridine-2-one LOX pharmacophore (**22**) exhibited dual COX and 5-LOX inhibition (COX-2 IC_50_ = 0.69 µM, COX-1 IC_50_ = 13.1 µM; 5-LOX IC_50_ = 5.0 µM) along with oral anti-inflammatory activity (ED_50_ = 27.7 mg/kg). In an elegant study, Chern and coworkers applied structure-based virtual screening and discovered a novel benzo[1.3.2]dithiazolium ylide-based ring scaffold as dual COX/5-LOX inhibitors [52]. The lead compound **23** (Figure 5), possessing a dithiazolium ylide 1,1-dioxide exhibited dual COX/5-LOX inhibition (COX-2 IC_50_ = 1.7 µM, COX-1 IC_50_ = 6.7 µM; 5-LOX IC_50_ = 1.22 µM). It was also interesting to note that **23** exhibited TNF-α inhibition (IC_50_ = 0.44 µM) in a lipopolysaccharide (LPS)-induced human acute monocytic leukemia cell line (THP-1) assay. The molecular modeling studies indicated that the (*S*)-enantiomer is potentially responsible for the observed COX-2 selectivity. *In vivo* anti-inflammatory activity data for these agents have not been reported.

## 5. Lp-PLA_2_ Inhibitors

The phospholipase A_2_ (PLA_2_) enzyme catalyzes the release of fatty acids such as AA, a critical rate-limiting step, by acting on membrane phospholipids (Figure 2). The released AA gets converted to various pro-inflammatory mediators such as prostaglandins, leukotrienes and platelet-activating factor (PAF) that are known to play a major role in regulating the vascular tone [53]. The PLA_2_ is classified into three major subtypes: secretory (sPLA_2_); cytosolic or Ca^2+^-activated (cPLA_2_); and inducible or Ca^2+^-independent (iPLA_2_). In this regard, Lp-PLA_2_ also known as platelet-activating factor acetylhydrolase (PAF-AH) is a Ca^2+^-independent PLA_2_ that is classified as group VIIA PLA_2_. Furthermore, recent studies have indicated that Lp-PLA_2_ is closely involved in the onset and progression of atherosclerosis [53,54,55,56,57]. The enzyme Lp-PLA_2 _ or PAF-AH (*EC 3.1.1.47*) was first identified from plasma that was known to hydrolyze/inactivate PAF, a phospholipid mediator produced from macrophages, monocytes, platelets and neutrophils involved in inflammatory diseases including atherosclerosis [58,59]. In humans, Lp-PLA_2_ is primarily produced from leukocytes and macrophages and is associated with circulating macrophages and low-density lipoproteins (LDL). It acts on polar phospholipids in oxidized LDL to form lysophosphatidylcholine and nonesterified phospholipids that are known to have proinflammatory properties by activating and recruiting macrophages/monocytes mediating plaque vulnerability, apoptosis, leading to onset and progression of atheroma [60,61]. These studies suggest that Lp-PLA_2_ is a unique biomarker to predict long-term cardiovascular risk [62,63,64]. 

**Figure 6:**
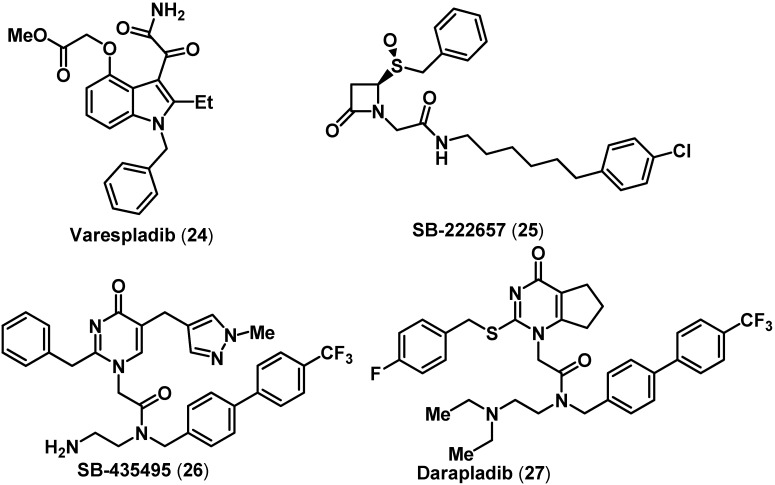
Chemical structures of some representative PLA_2_ inhibitors.

The drug discovery of novel small molecule PLA_2_ inhibitors is an ongoing effort [65,66]. Several indole-based inhibitors of sPLA_2_ have been developed to treat various inflammatory conditions such as pancreatitis, allergic rhinitis, rheumatoid arthritis, gout and atherosclerosis. For example, the indole derivative varespladib **24** (s-PLA_2_ IC_50_ = 15 nM, Figure 6) was developed as a treatment for rheumatoid arthritis and atherosclerosis [66,67,68]. A recent phase II trial showed that oral varespladib was able to reduce progression of atherosclerosis and associated cardiovascular events, without any evidence of adverse effects [67]. In this regard, a novel class of azetidinones represented by SB-222657 (**25**, Lp-PLA_2_ IC_50_ = 11.7 nM, Figure 6) were developed as active site directed Lp-PLA_2_ inhibitors [69]. Further studies led to the discovery of a potent Lp-PLA_2_ inhibitor SB-435495 possessing a pyrimidinone ring template (**26**, Lp-PLA_2_ IC_50_ = 0.06 nM, Figure 6) by GSK. Lead optimization resulted in the development of darapladib (**27**, Lp-PLA_2_ IC_50_ = 0.25 nM; Figure 6) as a clinical candidate and is the first agent developed as an Lp-PLA_2_ inhibitor to treat atherosclerosis and associated cardiovascular diseases [70,71]. Phase II studies of oral darapladib therapy led to reduced Lp-PLA_2_ activity in human atherosclerotic plaques in patients with stable coronary heart disease and reduced the levels of inflammatory mediator interleukin-6 [61,72,73,74]. Currently, darapladib is undergoing phase III trials. 

## 6. mPGES-1 Inhibitors

In the prostaglandin biosynthesis pathway, formation of the prostaglandin E_2_ (PGE_2_), a major mediator of pain and inflammation from prostaglandin H_2_ (PGH_2_) is catalyzed by PGE synthases such as cytosolic PGES (cPGES) and microsomal PGE synthases -1 and -2 (mPGES-1 and mPGES-2) [75,76,77,78,79]. Furthermore, mPGES-1 is known to transform COX-2 derived endoperoxides. In this regard, the membrane associated protein mPGES-1 is an inducible enzyme under inflammatory conditions such as RA, OA and atherosclerosis [77,78]. 

Although selective COX-2 inhibitors decrease the formation of proinflammatory PGE_2_, they exhibit cardiovascular side effects due their suppression of COX-2 derived vasodilatory prostacyclin (PGI_2_) biosynthesis [11,77,78]. Therefore, novel orally active small molecule mPGES-1 inhibitors are considered as an alternative strategy to develop anti-inflammatory agents with superior safety profile. Several ring templates including fatty acid derivatives have been developed as mPGES-1 and dual mPGES-1/5-LOX inhibitors [78]. 

**Figure 7:**
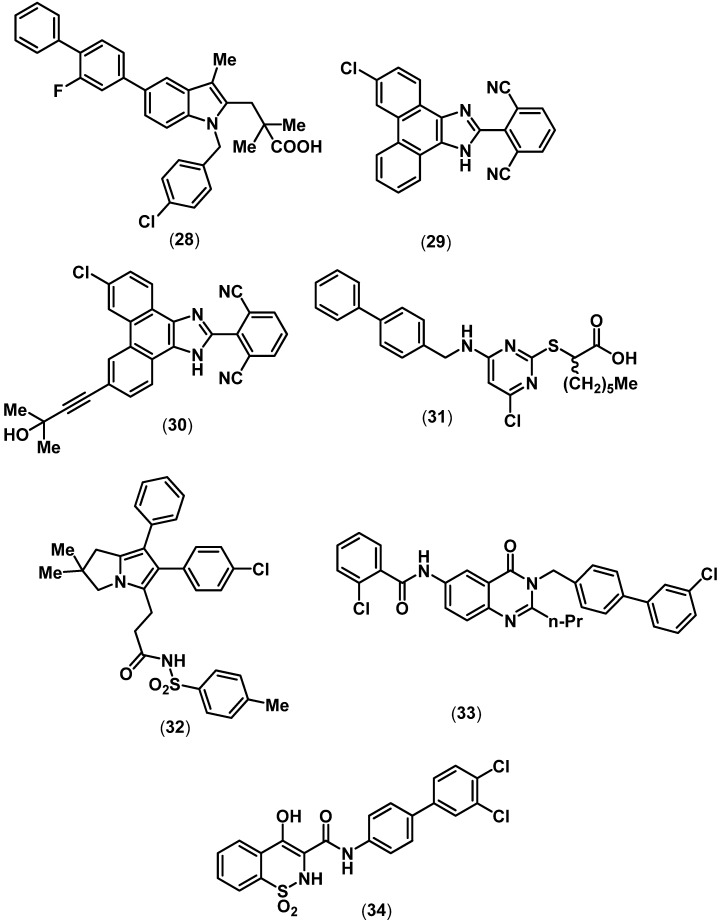
Chemical structures of some representative PLA_2_ inhibitors.

Merck Frosst reported the development of a number of novel ring templates as potent and selective mPGES-1 inhibitors [80,81,82]. An indole based agent (**28**, Figure 7), possessing a COOH group exhibited potent mPGES-1 inhibition (mPGES-1 IC_50_ = 0.007 µM) and effective PGE_2_ inhibition (IC_50_ = 8.0 µM) in cell based assays [80]. In another study, a series of phenanthrene imidazole template identified through a high-throughput (HTS) screening was developed as selective mPGES-1 inhibitors with oral activity [81].These agents lack an acidic (COOH) functional group. Compound **29** (Figure 7) was identified as a potent and selective mPGES-1 inhibitor (mPGES-1 IC_50_ = 0.001 µM; PGE_2_ inhibition IC_50_ = 0.42 µM) with *in vivo* analgesic activity, although **29** exhibited a short *in vivo* half life (t_1/2_ = 1.5 h) in animal models. In an extension of this work, a disubstituted phenanthrene imidazole (**30**, Figure 7) containing a propargylic tertiary alcohol moiety was developed that exhibited potent and selective mPGES-1 inhibition (mPGES-1 IC_50_ = 0.001 µM; mPGES-2 IC_50_ > 30 µM) and oral activity in lipopolysaccharide (LPS)-induced hyperalgesia guinea pig model (ED_50_ = 30 mg/kg). Interestingly, **30** exhibited a long *in vivo* pharmacokinetic properties in a rat model (t_1/2_ = 20 h) relative to **29** [82]. 

Recently, pirinixic acid derivatives were developed as a novel class of dual mPGES-1 and 5-LOX enzymes [83]. Among these group of agents **31** (Figure 7), a biphenyl pirinixic acid derivative, possessing a lipophilic *n*-hexyl chain along with a COOH group exhibited mPGES-1 inhibition (mPGES-1 IC_50_ = 1.3 µM; 5-LOX IC_50_ = 1.0 µM) along with moderate COX inhibition. It was also reported that enantiomeric forms of these agents exhibit similar activity profile. It should be noted that pirinixic acid ring template was initially reported as a peroxisome proliferator-activated receptor (PPAR-α) agonist [84,85]. Another elegant study developed dual mPGES-1/5-LOX agents based on an arylpyrrolizine ring template present in the anti-inflammatory agent licofelone [86]. The tolylsulfonimide **32** exhibited mPGES-1 inhibition (mPGES-1 IC_50_ = 2.1 µM) and moderate to no inhibition of enzymes COX-1 and COX-2 respectively. In this regard, Proschak and coworkers developed a group of nonacidic mPGES-1 inhibitors based on virtual library screening [87]. These studies identified a biphenyl quinazolinone ring template possessing a benzamide substituent (**33**), as an mPGES-1 inhibitor (IC_50_ = 0.5 µM) and did not exhibit COX-1/2 inhibition (IC_50_ > 30 µM). Furthermore, Pfizer Inc recently reported the development of selective mPGES-1 inhibitors derived from an oxicam ring template. In this regard the biphenyl oxicam derivative **34** (Figure 7), exhibited potent mPGES-1 inhibition (mPGES-1 IC_50_ = 0.016 µM) and relatively weak COX inhibition (COX-1 IC_50_ = 118 µM, COX-2 IC_50_ = 263 µM) [88].

## 7. TNF-α Inhibitors

**Figure 8:**
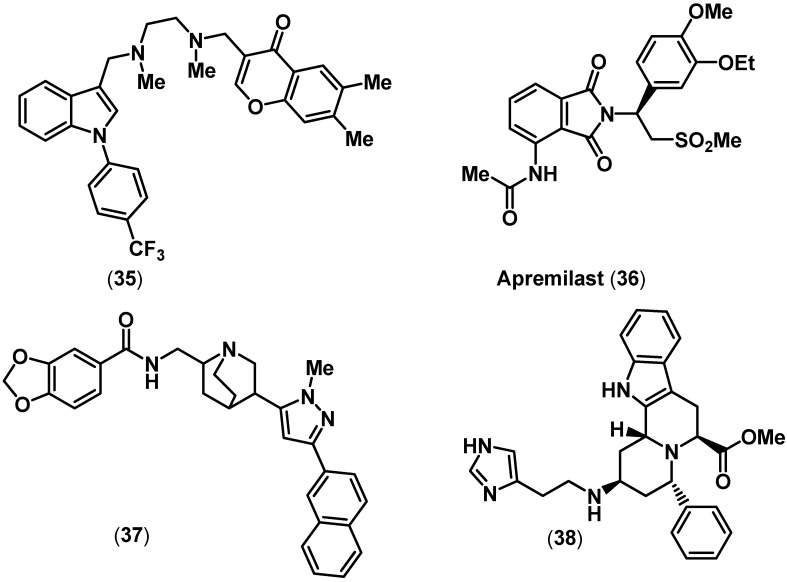
Examples of some representative TNF-α inhibitors.

Treating RA with biological therapeutics that target the proinflammatory cytokine TNF-α has been highly successful. However, their drawback includes high costs, lack of oral activity and adverse events such as autoimmune reactions [19,89,90,91]. In this regard several small molecule agents that inhibit TNF-α indirectly have been reported [19,23]. However, developing an orally active small molecule, that could act as a direct TNF-α inhibitor presents a significant challenge [90]. Toward this direction, scientists from Sunesis Pharmaceuticals Inc. developed a small molecule agent that possessed a phenyl indole and a chromone moiety (**35**, Figure 8). Compound **35** was promoting subunit disassembly of trimeric TNF-α and inhibition in both biochemical (TNF-α IC_50_ = 22 µM) and cell (TNF-α IC_50_ = 4.6 µM) based assays. In addition, the structure of **35** in complex with TNF-α dimer was solved [92]. Celgene Corporation recently developed apremilast (**36**, Figure 8) as an orally active dual phosphodiesterase-4 (PDE4) and TNF-α inhibitor that could be used to treat the autoimmune disorder psoriasis [93]. Compound **36**, has a dihydroisoindole ring template and is an (*S*)-enantiomer, whereas the corresponding (*R*) enantiomer of **36** was 5-fold less potent. In an LPS-induced cell based assay, **36** exhibited potent TNF-α inhibition (TNF-α IC_50_ = 0.077 µM) and oral activity in animal models (ED_50_ = 0.03 mg/kg). In addition, **36** exhibited potent PDE4 inhibition (PDE4 IC_50_ = 0.074 µM). Recently, Leung and coworkers reported two small molecules structurally similar to natural products. Structure-based design was used to identify small molecule TNF-α inhibitors [94]. Compound **37** was a quinuclidine derivative and **38** contained an indoloquinolizidine ring template (Figure 8). Both exhibited TNF-α inhibition (**37** TNF-α IC_50_ = 50 µM; **38** TNF-α IC_50_ = 22 µM), although in a cell based assay, **38** was less potent relative to **37**.

## 8. Conclusions

The story of treating fever, pain and inflammation continues to evolve. Small molecule NSAIDs have dominated the market for over a century. Advances in molecular biology, crystallography and rational drug design approaches have led to the successful identification of novel anti-inflammatory targets such as 5-LOX, COX-2, , Lp-PLA_2_, mPGES-1 and TNF-α, to mention a few. The risks involved in this endeavor, is clearly highlighted by the “coxib” controversy. In an era where new drug pipelines are drying-up and blockbuster agents are facing generic competition, the discovery of novel anti-inflammatory targets continues to propel the development of small molecule therapeutics to treat inflammatory conditions. It is evident that a rational drug discovery effort that combines HTS and fragment screening techniques can provide novel small molecule ring templates that can be optimized by medicinal chemistry methods, to exhibit suitable *in vivo* activity and optimal pharmacokinetic properties. In spite of the current increase in market share of biological therapeutics to treat inflammatory conditions, small molecule therapeutics continues to dominate the pharmaceutical landscape. The recent advances in deciphering the ability of small molecules to disrupt protein-protein interactions *in vivo*, provides an exciting opportunity to discover novel small molecule therapeutics to treat inflammation and a wide variety of disease states.
